# Finding the optimal mammography screening strategy: A cost‐effectiveness analysis of 920 modelled strategies

**DOI:** 10.1002/ijc.34000

**Published:** 2022-03-21

**Authors:** Lindy M. Kregting, Valérie D. V. Sankatsing, Eveline A. M. Heijnsdijk, Harry J. de Koning, Nicolien T. van Ravesteyn

**Affiliations:** ^1^ Department of Public Health Erasmus MC, University Medical Center Rotterdam Rotterdam The Netherlands

**Keywords:** breast cancer, cost‐effectiveness, mass screening, microsimulation modelling, screening strategies

## Abstract

Breast cancer screening policies have been designed decades ago, but current screening strategies may not be optimal anymore. Next to that, screening capacity issues may restrict feasibility. This cost‐effectiveness study evaluates an extensive set of breast cancer screening strategies in the Netherlands. Using the Microsimulation Screening Analysis‐Breast (MISCAN‐Breast) model, the cost‐effectiveness of 920 breast cancer screening strategies with varying starting ages (40‐60), stopping ages (64‐84) and intervals (1‐4 years) were simulated. The number of quality adjusted life years (QALYs) gained and additional net costs (in €) per 1000 women were predicted (3.5% discounted) and incremental cost‐effectiveness ratios (ICERs) were calculated to compare screening scenarios. Sensitivity analyses were performed using different assumptions. In total, 26 strategies covering all four intervals were on the efficiency frontier. Using a willingness‐to‐pay threshold of €20 000/QALY gained, the biennial 40 to 76 screening strategy was optimal. However, this strategy resulted in more overdiagnoses and false positives, and required a high screening capacity. The current strategy in the Netherlands, biennial 50 to 74 years, was dominated. Triennial screening in the age range 44 to 71 (ICER 9364) or 44 to 74 (ICER 11144) resulted in slightly more QALYs gained and lower costs than the current Dutch strategy. Furthermore, these strategies were estimated to require a lower screening capacity. Findings were robust when varying attendance and effectiveness of treatment. In conclusion, switching from biennial to triennial screening while simultaneously lowering the starting age to 44 can increase benefits at lower costs and with a minor increase in harms compared to the current strategy.

## Graphical abstract

What's new?

Since the implementation of breast cancer screening programs in Europe a few decades ago, breast cancer risk factors have increased and screening and treatment approaches have improved, potentially shifting the harm‐benefit balance of screening. This cost‐effectiveness study evaluates an extensive set of simulated breast cancer screening strategies in the Netherlands. Biannual screening for ages 40 to 76 is a cost‐effective strategy with more benefits than current screening (ages 50‐74). However, intensive screening strategies may not be achievable due to capacity constraints. Triennial screening for ages 44 to 71 or 44 to 74 increases benefits and reduces costs compared to the current screening strategy.

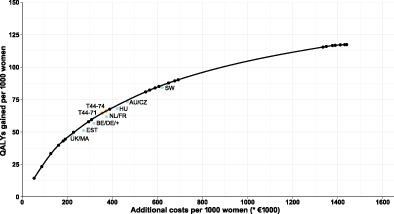

**Abbreviations**
BC: breast cancer
CISNET: Cancer Intervention and Surveillance Modelling Network
CvB: Centre for Population Screening
DCIS: ductal carcinoma in situ
ECIBC: European Commission Initiative on Breast Cancer
FP: false‐positive
IARC: International Agency for Research on Cancer
ICERs: incremental cost‐effectiveness ratios
LYG: life years gained
MISCAN‐Breast: MIcrosimulation SCreening ANalysis‐Breast
NETB: Dutch National Evaluation Team for Breast cancer screening
PPV: positive predictive value
RIVM: Dutch National Institute for Public health and Environment (Rijksinstituut voor Volksgezondheid en Milieu)
WTP: willingness to pay

## 1 INTRODUCTION

Breast cancer is the most prevalent cancer and leading cause of cancer‐related mortality amongst women worldwide.^1^ The first breast cancer screening programmes in Europe started in the late 1980s and have been shown to reduce breast cancer mortality significantly.^2^, ^3^ Currently, most European countries have implemented a screening programme, with some variety in starting and stopping ages and screening intervals.^3^ The cost‐effectiveness of these screening strategies has been proven in multiple studies.^4^ However, these studies only included a subset of possible alternative strategies.

An optimal screening strategy generates the best balance between benefits (eg, life years gained [LYG]) and harms (eg, overdiagnoses) at reasonable costs. Over the years, this balance between benefits and harms of breast cancer screening has been debated. Since implementation of screening, breast cancer risk factors increased and thereby the lifetime risk for women to be diagnosed with breast cancer increased.^5^, ^6^ It can be expected that this increased the population of women who benefit from breast cancer screening. In addition, both breast cancer screening and breast cancer treatment have improved (eg, digital mammography instead of film‐based mammography and more efficient adjuvant treatments), which has led to a decrease in breast cancer mortality.^7^, ^8^, ^9^ These changes might have shifted the harm‐benefit balance of breast cancer screening, implying that current screening strategies may not be optimal anymore.

The decision to implement a certain screening strategy is also based on the resources available. More than half of European countries face a limited capacity of screening due to a lack of human, physical or financial resources.^10^ This may lead to a maximum number of screening tests that can be performed in a country or region. This restriction can, in turn, decrease invitation coverage, narrow the age range of women invited, increase waiting time between tests and results or increase intervals between screening rounds.^11^, ^12^ Therefore, it is important to take capacity restrictions into account when possible changes in screening strategies are investigated.

In 1990, biennial breast cancer screening was implemented in the Netherlands for women ageing 50 to 69 years. This age range was extended to 74 in 1998. The current programme invites women aged 50 to 74 every 2 years. However, it is uncertain whether this remains the most optimal strategy. In addition, the Dutch breast cancer screening programme faces capacity issues which makes investigation of less intensive alternatives of interest and timely.^11^ Therefore, our study aimed to investigate the cost‐effectiveness of an extensive set of breast cancer screening strategies which differ in starting age, stopping age and screening interval.

## 2 MATERIALS AND METHODS

The effects of different screening strategies were predicted using the MIcrosimulation SCreening ANalysis‐Breast (MISCAN‐Breast) model.^7^ MISCAN‐Breast simulates individual life histories of women and, in a subset of them, the natural history of breast cancer. In addition, breast cancer screening programmes can be simulated to determine the effects of the screening protocol on breast cancer incidence, mortality and Quality Adjusted Life Years (QALYs). In the model, breast cancer starts with a preclinical ductal carcinoma in situ (DCIS) that can progress to invasive stages T1A, T1B, T1C and T2+, respectively. A tumour can become screen‐detected (in presence of screening), clinically detected (in presence of symptoms) or can progress to the next preclinical stage (Figure S1).^13^

### 2.1 Model parameters and assumptions

The MISCAN‐Breast model was updated with data on breast cancer treatment up to 2013 and previously calibrated for the natural history of breast cancer, and breast cancer survival rates with data up to 2015 (Supplement S1, p. 2).^14^

We simulated a cohort of 10 million women at average risk of developing breast cancer in which the tumour growth rate was distributed over a range including aggressive and slow growing rates. All women were born on the 1 January 1980 and life tables were based on data from Statistics Netherlands with a maximal life expectancy of 100 years.^15^ Outcomes were calculated for the women from age 40 until death. In order to calculate the full potential of the screening strategies, attendance rates were set at 100%.

The screening protocol in the model was adjusted for each investigated screening strategy. The intervals of interest were annual, biennial, triennial and quadrennial. Next to that, the start and stop ages were varied with a maximum of 10 years around the current screening ages in the Netherlands: starting age 40 to 60 and stopping age 64 to 84. This resulted in 920 different screening protocols, including no screening. Screening appointments were simulated to occur on the day the women reached the age at which an appointment was scheduled according to the protocol.

For each screening strategy, the number of invitations, screening mammograms, breast cancers detected by mode of detection (stage and age specific), total life‐years, life‐years with diagnosis (stage and age specific) and breast cancer deaths were predicted. From these predictions, number of breast cancer deaths averted, overdiagnoses, false positives, QALYs and additional costs were calculated.

### 2.2 Cost‐effectiveness analyses

A healthcare payer perspective was adopted and direct medical costs were calculated, including costs of screening, diagnostics and treatment. Data on costs and utilities were based on Geuzinge et al and indexed to 2018 (Supplement S1, p. 2 and Table 1).^16^ False positive (FP) findings were calculated using screen‐detected cancers from the model output and the positive predicted value (PPV) of recall. PPVs were specified by age (<50 and ≥50) and screening interval (Supplement S1, p. 2).

**TABLE 1:** Discounted model estimates of the number of breast‐cancer (BC) deaths averted, overdiagnoses, QALYs gained and additional costs (€) per 1000 women compared to no screening with percentage change compared to the current strategy (B50‐74)

Strategy	BC deaths averted		Overdiagnoses		False positives		QALYs gained		Additional costs (€)		ICER
Biennial 50‐74	4.9	—	5.8	—	89	—	61.7	—	374 762	—	Dominated
Quadrennial 60‐64	1.3	−75%	1.7	−72%	18	−80%	14.3	−77%	53 050	−86%	3699
Quadrennial 56‐64	1.9	−62%	2.2	−62%	25	−71%	23.3	−62%	87 100	−77%	3787
Quadrennial 52‐64	2.5	−50%	2.7	−54%	32	−64%	33.3	−46%	126 875	−66%	3974
Quadrennial 50‐66	3.0	−40%	3.2	−45%	38	−57%	39.8	−36%	161 450	−57%	5356
Quadrennial 50‐70	3.4	−32%	3.9	−33%	44	−50%	43.1	−30%	182 304	−51%	6327
Quadrennial 49‐69	3.4	−31%	3.8	−34%	51	−43%	44.4	−28%	191 039	−49%	6508
Quadrennial 47‐71	3.8	−23%	4.3	−25%	62	−30%	49.9	−19%	228 179	−39%	6856
Triennial 47‐71	4.4	−11%	4.9	−16%	83	−7%	58.0	−6%	294 724	−21%	8212
Triennial 46‐70	4.4	−11%	4.8	−17%	89	0%	59.6	−4%	307 927	−18%	8250
Triennial 44‐71	4.7	−4%	5.2	−11%	99	11%	64.6	5%	354 556	−5%	9321
Triennial 44‐74	5.0	1%	5.7	−3%	103	17%	66.2	7%	372 241	−1%	11 103
Triennial 43‐73	5.0	1%	5.6	−4%	109	23%	67.6	9%	388 503	4%	11 269
Biennial 43‐71	5.8	18%	6.1	5%	142	60%	80.9	31%	547 816	46%	11 963
Biennial 43‐73	6.0	22%	6.5	11%	146	65%	82.3	33%	565 623	51%	12 672
Biennial 42‐72	6.0	22%	6.4	10%	150	69%	84.0	36%	589 839	57%	14 502
Biennial 42‐74	6.2	26%	6.8	16%	154	74%	85.2	38%	606 977	62%	14 684
Biennial 41‐75	6.4	29%	7.0	21%	162	82%	87.8	42%	649 316	73%	16 162
Biennial 40‐74	6.4	30%	6.9	19%	165	86%	89.4	45%	676 927	81%	16 716
Biennial 40‐76	6.6	33%	7.3	25%	168	90%	90.2	46%	692 550	85%	19 164
Annual 40‐75	8.2	66%	8.6	47%	259	192%	115.5	87%	1 334 950	256%	25 478
Annual 40‐76	8.3	68%	8.8	50%	261	195%	116.0	88%	1 349 662	260%	29 090
Annual 40‐78	8.4	70%	9.1	57%	266	200%	116.7	89%	1 376 779	267%	39 319
Annual 40‐79	8.5	72%	9.3	60%	268	202%	116.8	89%	1 389 199	271%	63 919
Annual 40‐81	8.6	73%	9.7	66%	271	206%	117.2	90%	1 411 796	277%	65 630
Annual 40‐83	8.6	74%	10.0	71%	274	209%	117.3	90%	1 431 299	282%	132 200
Annual 40‐84	8.6	75%	10.1	73%	275	211%	117.4	90%	1 439 916	284%	133 050

*Note*: The table includes the current strategy and strategies on the efficiency frontier with corresponding ICERs. The strategy with a light grey background is not on the efficiency frontier, but included because it is the current strategy. The strategy with a dark grey background is the optimal strategy based on a WTP threshold of €20 000 per QALY gained. The other strategies with a grey background are candidate strategies based on more favourable QALYs and costs compared to the current strategy.

QALYs and costs were calculated for a situation with screening compared to no screening. Both effects and costs were discounted at 3.5% per year from 2020 to take time preferences into account.^17^

The screening strategies were ranked according to their costs (lowest to highest). Subsequently, incremental cost‐effectiveness ratios (ICERs) were calculated by dividing the difference in costs by the difference in QALYs between a strategy and its precursor in the ranking. Therefore, the ICER reflects the costs required to gain one QALY compared to the previous strategy. ICERs were not calculated for strategies that were dominated by another strategy (ie, another strategy gained more QALYs and required less costs). The ICERs were compared to a conservative willingness to pay (WTP) threshold of €20 000 per QALY gained.^18^ Strategies that did not exceed this threshold were considered to be cost‐effective.

### 2.3 International strategies

Within Europe, breast cancer screening programmes differ in ages covered and screening interval.^2^ The majority of these strategies were present in the set of 920 strategies which were modelled as part of the cost‐effectiveness analyses. In these model calculations, the screening strategies were applied to the situation in the Netherlands (ie, Dutch population size, screening parameters and breast cancer treatment effectiveness), preserving the assumption of 100% attendance. To evaluate the effectiveness of international strategies on the situation in the Netherlands, these strategies are plotted together with the efficiency frontier.

### 2.4 Capacity analyses

To evaluate the impact of different screening scenarios on screening capacity, the MISCAN‐Breast model was also used to simulate the effects of a subset of strategies of interest using a full population instead of a single cohort. The population that was simulated represented the Dutch female population based on population numbers until 2020 and population prognoses for the years after 2020.^19^ In this simulation, age‐specific participation rates were used as described in the sensitivity analyses. All other model parameters remained equal to the previous cohort simulations. The outcomes of interest produced by these population simulations was the average number of screens performed for the years 2020 to 2030.

### 2.5 Sensitivity analyses

Sensitivity analyses were performed using LYG as effect measure to calculate ICERs, age‐specific participation rates and assumptions on current adjuvant treatment use. The age‐specific participation rates were based on participation rates from the Dutch breast cancer screening programme between April 2017 and April 2019 (Table S2). Participation rates for ages below age 50 and after age 75 were extrapolated. Estimates for breast cancer treatment used between 2013 and 2020 were made based on trends in treatment changes between 2004 and 2011 (Table S3).

Additionally, separate ICERs were calculated for all strategies in which the starting age was at least 45, because the International Agency for Research on Cancer (IARC) found that there is sufficient evidence that breast cancer screening reduces mortality in women aged 50 to 74 and that the evidence for women aged 45 to 49 was nearly sufficient.^20^, ^21^

## 3 RESULTS

Without screening, the model estimated 149 breast cancer diagnoses and 49 breast cancer deaths per 1000 40‐year‐old women who were followed over their lifetime (no discount). Biennial screening for ages 50 to 74 (current strategy) was estimated to avert 16 breast cancer deaths (33%) and gain 231 QALYs per 1000 women compared to no screening. However, this strategy also led to 5 overdiagnoses and 187 FP screening results. When discounting, this would result in 61.7 QALYs gained and €374 763 additional costs, resulting in €6074 per QALY gained.

**FIGURE 1:**
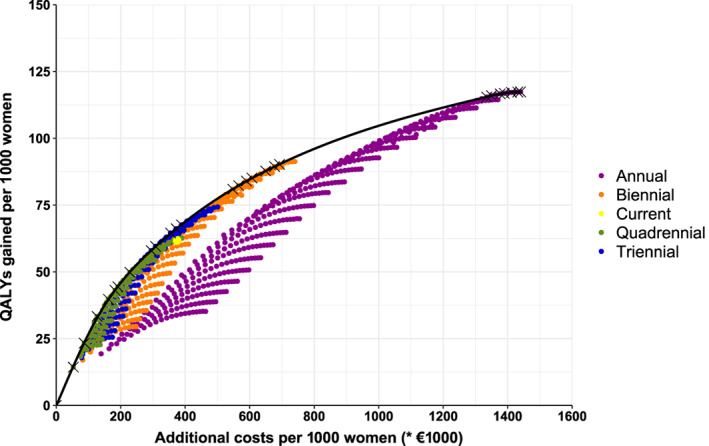
Cost‐effectiveness curve for scenarios with starting ages between 40 and 60 and stopping ages between 64 and 84, including efficiency frontier [Color figure can be viewed at wileyonlinelibrary.com]

Figure 1 shows the cost‐effectiveness curve of all investigated screening strategies (3.5% discounted). The efficiency frontier shows which strategies were efficient. Although the current strategy was close to the efficiency frontier, it was dominated. The efficiency frontier consisted of strategies with all four investigated screening intervals. However, all annual screening strategies were above the WTP threshold of €20 000.

### 3.1 Incremental cost‐effectiveness

Table 1 shows the model estimates for the current strategy and the screening strategies on the efficiency frontier. According to the conservative WTP threshold of €20 000 per QALY gained, biennial screening for the ages 40 to 76 would be the preferred strategy with an ICER of €19 164 per QALY gained (3.5% discounted). This strategy resulted in more breast cancer deaths averted (6.6 vs 4.9 per 1000 women) and more QALYs gained (90.2 vs 61.7) than current screening. On the other hand, there were more overdiagnoses, (7.3 vs 5.8 per 1000 women), many more false positives (168 vs 89) and additional costs were higher (€692 550 vs €374 763).

To achieve at least the same number of QALYs as the current strategy, triennial screening for the ages 44 to 71 was the first strategy on the frontier. This strategy gained more QALYs than the current strategy (64.6 vs 61.7 per 1000 women) and had lower additional costs (€354 556 vs €374 763). This resulted in an ICER of €9321 per QALY gained. In addition, the number of overdiagnoses was lower (5.2 vs 5.8 per 1000 women) while the number of false positives was higher (99 vs 89) compared to the current strategy. Another strategy of interest could be triennial screening for the ages 44 to 74. The additional costs of this strategy were approximately the same as the current strategy (€372 241 vs €373 763 per 1000 women), while the amount of QALYs gained increased (61.7 vs 66.2), the number of overdiagnoses was slightly lower (5.7 vs 5.8) and the number of false positives was higher (103 vs 89). In this strategy, the ICER was estimated to be €11 103 per QALY gained.

### 3.2 International strategies

**FIGURE 2:**
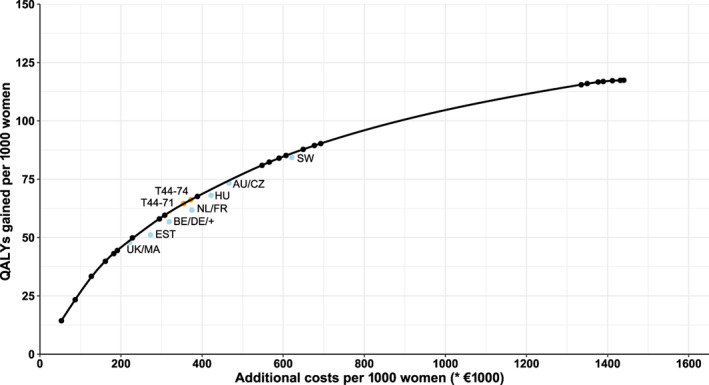
Effects of internationally implemented strategies assuming 100% attendance. The United Kingdom (UK) and Malta (MA) triennial 50 to 69; Estonia (EST) and biennial 50 to 64; Belgium (BE), Germany (DE), Poland, Cyprus, Denmark, Finland, Latvia, Lithuania, Luxembourg, Norway, Poland, Slovenia and Switzerland biennial 50 to 69; the Netherlands (NL) and France (FR) biennial 50 to 74; Hungary (HU) biennial 45 to 65; Austria (AU) and Czech republic (CZ) biennial 45 to 69; Sweden (SW) biennial 40 to 69 [Color figure can be viewed at wileyonlinelibrary.com]

Figure 2 shows the effects of breast cancer screening strategies implemented by different European countries if they would be implemented in the Netherlands together with the efficiency frontier from Figure 1. None of the internationally implemented strategies were on the efficiency frontier, although all strategies were very close.

### 3.3 Capacity analyses

Population simulations were performed for the strategies biennial 50 to 74, biennial 40 to 76, triennial 44 to 71 and triennial 44 to 74. Biennial screening for ages 40 to 76 was estimated to result in 34% more screens being performed per year compared to the current screening strategy (Table 2). Triennial screening for ages 44 to 71 or for ages 44 to 74 would lead to a reduction in the number of screens performed of 22% and 17%, respectively.

**TABLE 2:** Number of screens performed per year for the strategies of interest

	# screens per year	% difference compared to biennial 50‐74	ICER^a^
Biennial 50‐74	1 057 896	—	Dominated
Biennial 40‐76	1 416 427	+34%	19 164
Triennial 44‐71	820 636	−22%	9321
Triennial 44‐74	880 759	−17%	11 103

^a^ The ICERs were obtained from Table 1 and were based on calculations including all strategies on the efficiency frontier.

### 3.4 Sensitivity analyses

When looking at LYG as effective measure instead of QALYs gained, the same three strategies were of interest (Table S4). In all three strategies the amount of LYG was increased compared to the amount of LYG in the current strategy.

Taking into account age‐specific attendance rates in all modelled screening strategies decreased the number of breast cancer deaths averted, overdiagnoses, false positives, QALYs gained and additional costs (Table 3). This also slightly changed which strategies were present on the efficiency frontier and the accompanying ICERs (Supplement S1, p. 7).

**TABLE 3:** Discounted model estimates for sensitivity analyses using age‐dependent attendance rates on the number of breast‐cancer (BC) deaths averted, overdiagnoses, QALYs gained and additional costs (€) per 1000 women compared to no screening with percentage change compared to the current strategy (B50‐74)

Strategy	BC deaths averted		Overdiagnoses		False positives		QALYs gained		Additional costs (€)		ICER
Biennial 50‐74	4.1	—	4.8	—	73	—	51.0	—	293 414	—	Dominated
Quadrennial 60‐64	1.0	−76%	1.3	−73%	14	−81%	11.5	−77%	41 468	−86%	3611
Quadrennial 56‐64	1.5	−63%	1.8	−63%	20	−72%	18.9	−63%	68 621	−77%	3652
Quadrennial 52‐64	2.0	−52%	2.1	−55%	25	−66%	26.3	−48%	99 199	−66%	4117
Quadrennial 51‐67	2.3	−42%	2.6	−45%	30	−58%	30.7	−40%	121 794	−58%	5131
Quadrennial 51‐71	2.6	−35%	3.1	−35%	35	−52%	33.1	−35%	136 537	−53%	6196
Quadrennial 50‐70	2.7	−34%	3.1	−35%	35	−52%	34.2	−33%	143 159	−51%	6339
Quadrennial 47‐71	3.0	−27%	3.4	−29%	48	−34%	38.9	−24%	175 861	−40%	6900
Triennial 48‐69	3.2	−21%	3.5	−26%	60	−17%	43.2	−15%	205 425	−30%	6963
Triennial 48‐72	3.5	−15%	3.9	−17%	65	−11%	45.0	−12%	219 527	−25%	7701
Triennial 45‐72	3.7	−8%	4.1	−13%	77	6%	49.9	−2%	263 080	−10%	8904
Biennial 44‐72	4.7	15%	5.0	6%	109	49%	63.6	25%	406 206	38%	10 404
Biennial 44‐74	4.8	18%	5.3	12%	112	54%	64.6	27%	418 729	43%	13 073
Biennial 43‐73	4.8	19%	5.3	11%	120	65%	65.9	29%	436 779	49%	13 668
Biennial 43‐75	5.0	22%	5.5	16%	123	69%	66.8	31%	448 488	53%	14 007
Biennial 42‐74	5.0	23%	5.5	15%	121	66%	68.0	33%	466 350	59%	14 627
Biennial 40‐74	5.1	27%	5.6	17%	128	76%	71.2	40%	516 777	76%	15 710
Biennial 40‐76	5.3	29%	5.9	23%	131	80%	71.8	41%	527 777	80%	17 784
Annual 42‐73	6.5	60%	6.8	42%	197	171%	90.6	78%	898 319	206%	19 719
Annual 42‐74	6.6	62%	6.9	46%	199	174%	91.1	79%	910 480	210%	21 896
Annual 42‐75	6.7	64%	7.1	49%	202	177%	91.7	80%	921 916	214%	21 997
Annual 41‐73	6.6	63%	6.8	44%	204	181%	92.8	82%	947 557	223%	22 208
Annual 41‐75	6.8	67%	7.2	51%	209	187%	93.9	84%	971 196	231%	22 306
Annual 40‐75	6.9	69%	7.3	53%	216	197%	96.1	88%	1 022 070	248%	23 364
Annual 40‐77	7.0	72%	7.6	60%	220	202%	96.8	90%	1 042 553	255%	28 570
Annual 40‐81	7.2	76%	8.2	72%	226	210%	97.5	91%	1 075 099	266%	43 734
Annual 40‐82	7.2	77%	8.3	74%	227	212%	97.6	91%	1 081 621	269%	80 786
Annual 40‐84	7.2	78%	8.5	78%	229	214%	97.7	92%	1 092 504	272%	174 927

*Note*: The table includes the current strategy and strategies on the efficiency frontier with corresponding ICERs. The strategy with a light grey background is not on the efficiency frontier, but included because it is the current strategy. The strategy with a dark grey background is the optimal strategy based on a WTP threshold of €20 000 per QALY gained. The other strategies with a grey background are candidate strategies based on more favourable QALYs and costs compared to the current strategy.

When alternative assumptions on current adjuvant treatment use were used, the number of breast cancer deaths averted, overdiagnoses, false positives and QALYs gained were slightly lower (Table S5). On the other hand, the additional costs slightly increased. In addition, the strategies which were present on the efficiency frontier and the accompanying ICERs slightly changed compared to the base case analyses (Supplement S1, p. 7).

If only strategies with starting ages between age 45 and 60 were included in the ICER calculations, different strategies appear on the efficiency frontier (Table 4). When considering the conservative WTP threshold of €20 000 per QALY gained, biennial screening for ages 45 to 75 would be optimal (ICER: 17 147). When comparing to the current strategy, the triennial 45 to 72 strategy would gain more QALYs (63.0 vs 61.7 per 1000 women) and have less additional costs (€340 815 vs €374 763).

**TABLE 4:** Discounted model estimates for sensitivity analyses with starting ages ranging from age 45 to age 60 on the number of breast cancer (BC) deaths averted, overdiagnoses, QALYs gained and additional costs (€) per 1000 women compared to no screening with percentage change compared to the current strategy (B50‐74)

Strategy	BC deaths averted		Overdiagnoses		False positives		QALYs gained		Additional costs (€)		ICER
Biennial 50‐74	4.9	—	5.8	—	89	—	61.7	—	374 762	—	Dominated
Quadrennial 60‐64	1.3	−75%	1.7	−72%	18	−80%	14.3	−77%	53 050	−86%	3699
Quadrennial 56‐64	1.9	−62%	2.2	−62%	25	−71%	23.3	−62%	87 100	−77%	3787
Quadrennial 52‐64	2.5	−50%	2.7	−54%	32	−64%	33.3	−46%	126 875	−66%	3974
Quadrennial 50‐66	3.0	−40%	3.2	−45%	38	−57%	39.8	−36%	161 450	−57%	5356
Quadrennial 50‐70	3.4	−32%	3.9	−33%	44	−50%	43.1	−30%	182 304	−51%	6327
Quadrennial 49‐69	3.4	−31%	3.8	−34%	51	−43%	44.4	−28%	191 039	−49%	6508
Quadrennial 47‐71	3.8	−23%	4.3	−25%	62	−30%	49.9	−19%	228 179	−39%	6856
Triennial 47‐71	4.4	−11%	4.9	−16%	83	−7%	58.0	−6%	294 724	−21%	8212
Triennial 46‐70	4.4	−11%	4.8	−17%	89	0%	59.6	−4%	307 927	−18%	8250
Triennial 45‐72	4.7	−5%	5.3	−10%	100	12%	63.0	2%	340 815	−9%	9620
Biennial 45‐71	5.5	12%	5.9	1%	128	45%	75.3	22%	485 671	30%	11 765
Biennial 45‐73	5.7	16%	6.3	8%	133	50%	76.7	24%	503 538	34%	12 763
Biennial 45‐75	5.9	19%	6.6	14%	137	54%	77.6	26%	519 870	39%	17 147
Biennial 45‐77	6.0	21%	7.0	20%	140	58%	78.3	27%	534 735	43%	21 629
Annual 45‐74	7.3	47%	7.7	32%	205	131%	97.9	58%	993 630	165%	23 500
Annual 45‐76	7.4	50%	8.1	39%	210	137%	98.9	60%	1 023 878	173%	29 174
Annual 45‐77	7.5	52%	8.3	43%	213	140%	99.3	61%	1 037 823	177%	35 320
Annual 45‐78	7.6	53%	8.5	46%	215	142%	99.6	61%	1 050 976	180%	44 453
Annual 45‐80	7.6	55%	8.8	52%	219	147%	100.0	62%	1 075 080	187%	53 111
Annual 45‐81	7.7	55%	9.0	55%	220	148%	100.1	62%	1 085 994	190%	127 467
Annual 45‐84	7.8	57%	9.5	62%	224	153%	100.3	62%	1 114 172	197%	136 768

*Note*: The table includes the current strategy and strategies on the efficiency frontier with corresponding ICERs. The strategy with a light grey background is not on the efficiency frontier, but included because it is the current strategy. The strategy with a dark grey background is the optimal strategy based on a WTP threshold of €20 000 per QALY gained. The other strategies with a grey background are candidate strategies based on more favourable QALYs and costs compared to the current strategy.

## 4 DISCUSSION

This is the first study to investigate the cost‐effectiveness of an extensive set of breast cancer screening strategies varying in starting age, stopping age and screening intervals. Using a conservative WTP threshold of €20 000 per QALY gained, biennial screening for ages 40 to 76 was preferred. This strategy resulted in more breast cancer deaths averted and QALYs gained than current screening. However, it required a 34% higher screening capacity per year than the current strategy. When taking into account capacity issues, less intensive alternative cost‐effective strategies with comparable costs or QALYs as the current strategy were triennial screening for the ages 44 to 71 or 44 to 74. Thus, our results indicate that triennial screening with a lower starting age is a very good alternative, especially for countries facing capacity issues, because it can lead to more benefits at similar costs. We acknowledge that starting screening earlier is controversial, in particular before the age of 45, due to lack of evidence of screening effectiveness. Therefore, sensitivity analyses were performed in which the starting age of screening was at least 45 which resulted in triennial screening for the ages 45 to 72 to be a good alternative to current screening.

Our study had some limitations. One is the need to make assumptions, because it remains largely unknown how some parameters change for different screening intervals and for a population of women under 50 and over 74.^2^, ^22^ Especially the screening sensitivity, effectiveness and PPV for women under the age of 45 is largely unknown.^20^ Therefore, we assumed these factors to be the same as for women aged 45 to 50. Benefits (LYG) in young women might be higher due to longer remaining life expectancy, but harms (FPs) larger, due to higher breast density.^23^ We assumed the PPV for women under the age of 50 to be lower than in women over 50. However, since the PPV in the Netherlands is relatively high compared to other countries, we expect that in countries with a lower PPV, screening for younger ages may be less favourable.^24^ Another limitation is the choice to not include hybrid screening strategies (ie, different intervals for different age groups). Combining different intervals by age group may lead to a better harm‐benefit balance for each specific age group. However, including hybrid strategies in the analyses would lead to a major increase in possible strategies.

The chosen WTP threshold of €20 000 per QALY gained is conservative compared to other studies which use WTP thresholds of €30 000 per QALY gained or more.^4^, ^25^, ^26^, ^27^ However, even when using this conservative threshold, already a rather intensive screening strategy was found to be optimal, with increased harms and a higher required capacity than the current screening strategy. Using a WTP threshold of €30 000 will indicate more intensive screening strategies to be optimal (ie, annual screening for ages 40‐76) with more harms and a higher required capacity. Another assumption we made was 100% screening attendance. Even though an attendance rate of 100% is practically impossible and ethically undesirable, this assumption made it possible to estimate the potential of each screening strategy and it allowed for a comparison to the literature. Another reason to model 100% attendance was to prevent the preferred strategy to be too intensive for women who choose to comply completely. To estimate more realistic effects of the screening strategies, sensitivity analyses were performed with Dutch age‐specific attendance rates. Attendance rates may differ between different screening intervals, however, a lack of participation estimates for different screening intervals made it impossible to incorporate this in the sensitivity analyses. In addition, screening appointments were assumed to take place on the day the women reached the age at which an appointment was planned according to the strategy instead of spread over the interval between screening ages. Accordingly, the average screening age will slightly increase if a modelled strategy is implemented.

Strengths of our study include the use of a well‐established, calibrated, model and the evaluation of an extensive set of scenarios. So far, most cost‐effectiveness studies on breast cancer screening have only investigated the effects of a restricted number of screening strategies. For example, a study in Spain modelled 20 strategies which found that starting screening at age 50 is preferred over age 40 or 45, and stopping at age 74 is preferred over age 69.^28^ Furthermore, they concluded that the ICER was much higher for annual than for biennial strategies. However, our study only included annual and biennial strategies, which could have led to underestimation of ICERs.^29^ A Slovenian modelling study investigated the cost‐effectiveness of 36 breast cancer screening strategies and found that screening triennially for the ages 40 to 80 would be optimal (ICER: €13 352 per QALY gained).^26^ However our study included intervals of a maximum of 3 years, which possibly omitted efficient strategies with longer intervals.^29^ Next to that, an American study modelled the cost‐effectiveness of 66 strategies including 5‐year intervals, however, 4‐year intervals were left out, which may cause a kinked efficiency frontier.^29^, ^30^ Our study did not select an optimal strategy, however, the calculated ICERs were much higher than ICERs of comparable strategies in our analysis. Another Spanish study did investigate an extensive set of 2.625 screening strategies in which 24 strategies were uniform for the total population and 2601 were risk‐based.^31^ Our study found risk‐based screening to be more efficient than uniform strategies. They proposed risk‐based screening including 3 and 5 year intervals for low risk groups and annual screening for high risk groups. However, just like the American study, 4‐year intervals were not investigated. Next to that, all four studies only included round starting and stopping ages (40, 45, 50, etc.) which results in a subset of possible screening strategies. Although, for implementation round numbers seem more logical and feasible, by only investigating a subset of strategies the calculations of the ICERs can be incomplete. This could lead to misidentification of an inefficient strategy as optimal.^29^

The breast cancer screening strategies that are currently implemented in the Netherlands and in other European countries were not on the efficiency frontier. This raises the question of why they were implemented. Most screening programmes were implemented more than 20 years ago. Back then, many countries based their decisions mainly on randomised controlled trials instead of extensive cost‐effectiveness studies. Partly because it was not possible to calculate or simulate the effects of a large set of strategies because of the computational power. Also, although there are still uncertainties about screening in women before the age of 50, more knowledge has been gained in the last decades. This is also partly reflected in the newest breast cancer guidelines on screening ages and frequencies by the European Commission Initiative on Breast Cancer (ECIBC) which suggests no screening between the ages 40 to 44, biennial or triennial screening for the ages 45 to 49, biennial screening for the ages 50 to 69 and triennial screening for the ages 70 to 74.^32^ These recommendations, however, did not consider cost‐efficiency nor resources and capacity. On top of these methodological issues, changes in breast cancer incidence, screening modalities and treatment options may have shifted optimal screening towards triennial intervals and starting at younger ages becoming more beneficial. Although, differences in incidence, treatment and population characteristics between countries could lead to different strategies being optimal than the ones found in the current study using the Dutch situation. Furthermore, the strategies that were present on the efficiency frontier are currently not implemented anywhere else. Which means that there is no information yet on the true effectiveness of these strategies when implemented.

Although performed from a Dutch perspective, our findings might be relevant for other countries as well, especially those facing capacity issues.^10^, ^11^, ^12^ The COVID‐19 pandemic has led to a disruption or restriction in breast cancer screening in many countries, raising the question of how to restart screening programmes.^33^ Combined with capacity limitations, this may encourage policy makers to consider programme changes. Our study can inform these policy makers about the cost‐effectiveness and some of the benefits and harms of alternative screening strategies. The alternative triennial screening strategies were estimated to require a lower screening capacity than the current Dutch biennial screening programme. Implementing one of these strategies in the Netherlands can be expected to reduce the personnel capacity problems reported by the Dutch National Institute for Public health and Environment (RIVM).^11^ However, before a new programme can be implemented, additional factors need to be taken into account, such as screening harms, logistical factors, population equity and level of acceptance by the target population. Furthermore, the time and costs of the implementation processes need to be considered. By measuring QALYs, the effects of multiple harms were incorporated; however policy makers may weigh certain harms differently. The triennial strategies were estimated to slightly decrease the amount of overdiagnosis, the amount of false positives increased and one of the strategies was estimated to avert fewer breast cancer deaths than the current strategy. Furthermore, triennial strategies may lead to more interval cancers than biennial screening strategies.

In conclusion, we found that the current Dutch breast cancer screening strategy and strategies applied in many other European countries were not the most cost‐effective options and can be improved. A quite more intensive screening scenario of screening biennially for the ages 40 to 76 years was found to be optimal. More realistically, restricting the costs and the number of screens, we found that triennial screening for ages 44 to 71 or 44 to 74 would be the preferred breast cancer strategy.

## CONFLICT OF INTEREST

The authors have declared no conflicts of interest.

## AUTHOR CONTRIBUTIONS

Lindy M. Kregting: Conceptualisation, data curation, formal analysis, investigation, methodology, validation, visualisation, writing—original draft. Valerie D. V. Sankatsing: Conceptualisation, data curation, methodology, writing—review & editing. Eveline A. M. Heijnsdijk: Conceptualisation, data curation, validation, writing—review & editing. Harry J. de Koning: Conceptualisation, funding acquisition, supervision, validation, writing—review & editing. Nicolien T. van Ravesteyn: Conceptualisation, data curation, funding acquisition, methodology, supervision, validation, writing—review & editing. The work reported in the article has been performed by the authors, unless clearly specified in the text.

## Supporting information


**Appendix S1** Supporting Information.

## Data Availability

Data used as input for the MISCAN‐Breast model can be requested from the primary source (Table S1). Model outcome data can be made available upon reasonable request.
