# Supplementary material for: Finding the optimal mammography screening strategy: A cost‐effectiveness analysis of 920 modelled strategies
Source: Int J Cancer. 2022 Mar 21;151(2):287–96. doi: 10.1002/ijc.34000 (PMC9310858; doi:10.1002/ijc.34000)
Supplement: Supplementary file 1 — Appendix S1 Supporting Information. [file IJC-151-287-s001.pdf]

Finding the optimal mammography screening strategy: a cost-effectiveness analysis of 920 modelled strategies

Lindy M. Kregting, Valerie D.V. Sankatsing, Eveline A.M. Heijnsdijk, Harry J. de Koning, Nicolien T. van Ravesteyn

Appendix

Index

Appendix figure 1 MISCAN-Breast transitions 1

Appendix page 2 MISCAN parameters 2

Appendix table 1 Price and utility parameters 3

Appendix table 2 Age-specific participation rates 3

Appendix table 3 Treatment parameter estimates 5

Appendix table 4 Model estimates of the number of life years gained 6

Appendix page 7 Results sensitivity analyses 7

Appendix table 5 Model estimates using alternative treatment assumptions 8

References 9

Appendix figure 1

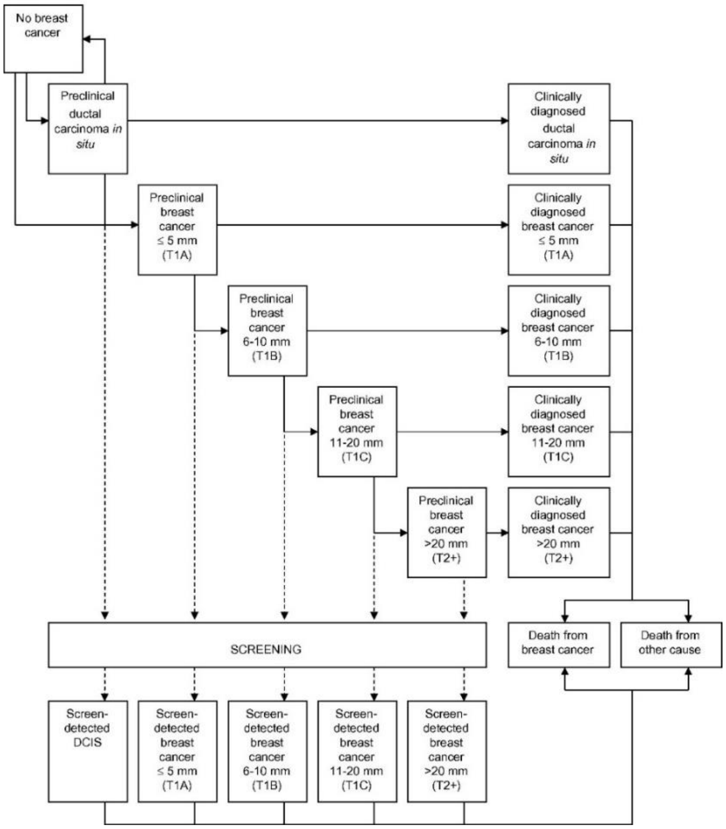

Appendix figure 1 MISCAN-Breast transitions (de Gelder et al. (2009)[1])

Appendix page 2

Calibration of MISCAN-Breast

During the recent calibration or MISCAN-Breast, stage- and age-specific sensitivity of digital mammography, breast cancer background incidence, stage- and age-specific mean duration of preclinical screen-detectable breast cancer and progression and regression of rate of DCIS were recalibrated.[2] This was done using data from the Dutch breast cancer screening programme on interval cancers (2004-2011), screen-detected cancers (2004-2013), and stage distribution at detection and data from the Netherlands Comprehensive Cancer Organisation (IKNL) on age-specific breast cancer incidence between 1975 and 2013. These calibrations led to model predictions that complied with Dutch breast cancer and breast cancer screening data and trends. Furthermore, the probabilities of receiving adjuvant treatment (no adjuvant treatment, hormonal therapy, chemo therapy, or combination therapy) were updated using data from IKNL over the years 2004-2013.[3]

Stage specific and age specific cure and survival rates after screen-detection were based on the Swedish randomised controlled trials.[4-7] Survival rates were also specified by lymph node status. The parameters were based on Dutch population data from Statistics Netherlands and IKNL complemented with data from meta-analyses from EBCTCG.[3,8,9]

Cost-effectiveness analyses

Diagnostic and treatment costs were specified per tumour stage. Data on costs and utilities were based on Geuzinge et al. and indexed to 2018 (Appendix Table 1).[10] Additionally, costs on biopsies were combined (€293.22) and costs of screening mammograms (€68.97) were updated based on the Dutch breast cancer screening monitor 2017-2018.[11] Furthermore, the costs of the last year of life before a breast cancer death compared to an other-cause death were updated with calculations based on data from Polder et al and Bakx et al., indexed to 2018 (€12,367.88, see appendix table 1).[12,13] QALYs were calculated by applying utility decrements on the average utility in Dutch women (0.858).[14] Utility decrements were used for screening participation (0.006), referral (0.105), life years of breast cancer treatment (stage specific), and cause of death (breast cancer or other causes).

FP findings were calculated using screen-detected cancers from the model output and the positive predicted value (PPV) of recall. PPVs were specified by age (<50 and ≥50) and screening interval. Biennial screening PPVs used were 12% for women <50, and 28% for women ≥50.[15-17] Ratios of the PPVs for annual and triennial screening were calculated based on findings in the U.S. Norway, and Spain.[18] Ratios for the PPVs for quadrennial screening were extrapolated based on the ratios for annual and triennial screening. These ratios were used to estimate PPVs for annual, triennial and biennial screening in the Netherlands. This resulted in PPVs of 10% (<50) and 24% (≥50) for annual screening, 13% (<50) and 31% (≥50) for triennial screening, and 15% (<50) and 35% (≥50) for quadrennial screening.[2]

Appendix table 1

Appendix table 1 Price and utility parameters

Unit prices		
**Procedure**	**Price* (€)**	**Source**
Screening invitation	2	Sankatsing et al. [19]
Mammography (in screening setting)	68.97	IKNL monitor [11]
Mammography (in hospital setting)	91.97	Geuzinge et al.[10]
MRI	272	Geuzinge et al.[10]
Palpation	72.57	Geuzinge et al.[10]
Ultrasound	115.23	Geuzinge et al.[10]
FNA	293.22	Erasmus MC; CZ tariff tool [20]
Biopsy	293.22	Erasmus MC; CZ tariff tool [20]
GP consultation	17.69	Geuzinge et al.[10]
GP consultation (telephone)	34.34	Geuzinge et al.[10]
**Treatment costs according to T-stage**		
DCIS	5520	Geuzinge et al.[10]
T1a, N-	6376	Geuzinge et al.[10]
T1a, N+	6617	Geuzinge et al.[10]
T1b, N-	7441	Geuzinge et al.[10]
T1b, N+	10110	Geuzinge et al.[10]
T1c, N-	9073	Geuzinge et al.[10]
T1c, N+	9901	Geuzinge et al.[10]
T2+, N-	8480	Geuzinge et al.[10]
T2+, N-	8448	Geuzinge et al.[10]
Palliative therapy (last year of life) of breast cancer patients compared to palliative care for other causes of death	12,367.88	Calculations based on Polder et al. and Bakx et al. [12,13]
**Health state**	**Utility and duration**	**Source**
No breast cancer	0.858	Versteegh et al. [14]
Undergoing screening	0.006 (disutility) for 1 week	De Haes et al. [21]
Referral	0.105 (disutility) for 5 weeks	De Haes et al. [21]
DCIS/localised breast cancer	0.772 for 2 years	Stout et al. [22]
Regional breast cancer	0.644 for 2 years	Stout et al. [22]
Metastasis	0.515 until death	Stout et al. [22]
Death	0	

*All prices were indexed to 2018 using consumer price indices.

Appendix table 2

Appendix table 2 Age-specific participation rates

**Age**	**Participation rate (%)**	**Source**
40	69.66	Linear extrapolation based on age 50-65

41	70.04	Linear extrapolation based on age 50-65
42	70.42	Linear extrapolation based on age 50-65
43	70.80	Linear extrapolation based on age 50-65
44	71.18	Linear extrapolation based on age 50-65
45	71.56	Linear extrapolation based on age 50-65
46	71.94	Linear extrapolation based on age 50-65
47	72.32	Linear extrapolation based on age 50-65
48	72.71	Linear extrapolation based on age 50-65
49	73.09	Linear extrapolation based on age 50-65
50	73.46	Dutch participation data from April 2017 till April 2019
51	74.09	Dutch participation data from April 2017 till April 2019
52	74.25	Dutch participation data from April 2017 till April 2019
53	74.63	Dutch participation data from April 2017 till April 2019
54	74.69	Dutch participation data from April 2017 till April 2019
55	75.02	Dutch participation data from April 2017 till April 2019
56	75.87	Dutch participation data from April 2017 till April 2019
57	75.97	Dutch participation data from April 2017 till April 2019
58	77.07	Dutch participation data from April 2017 till April 2019
59	77.02	Dutch participation data from April 2017 till April 2019
60	76.94	Dutch participation data from April 2017 till April 2019
61	77.53	Dutch participation data from April 2017 till April 2019
62	78.29	Dutch participation data from April 2017 till April 2019
63	78.54	Dutch participation data from April 2017 till April 2019
64	78.83	Dutch participation data from April 2017 till April 2019
65	79.03	Dutch participation data from April 2017 till April 2019
66	79.00	Dutch participation data from April 2017 till April 2019
67	78.80	Dutch participation data from April 2017 till April 2019
68	78.95	Dutch participation data from April 2017 till April 2019
69	78.45	Dutch participation data from April 2017 till April 2019
70	77.93	Dutch participation data from April 2017 till April 2019
71	77.61	Dutch participation data from April 2017 till April 2019
72	76.09	Dutch participation data from April 2017 till April 2019
73	74.62	Dutch participation data from April 2017 till April 2019
74	73.24	Dutch participation data from April 2017 till April 2019
75	71.10	Dutch participation data from April 2017 till April 2019
76	69.78	Linear extrapolation based on age 71-75
77	68.19	Linear extrapolation based on age 71-75
78	66.60	Linear extrapolation based on age 71-75
79	65.02	Linear extrapolation based on age 71-75
80	63.43	Linear extrapolation based on age 71-75
81	61.84	Linear extrapolation based on age 71-75
82	60.26	Linear extrapolation based on age 71-75
83	58.67	Linear extrapolation based on age 71-75
84	57.08	Linear extrapolation based on age 71-75

Appendix table 3

Appendix table 3 Averages of recent treatment estimates by expert opinion. Stratified by mode of detection, tumour stage at diagnosis and age categories.

**Screen detected breast cancers**									
	**DCIS**	**T1AN-**	**T1AN+**	**T1BN-**	**T1BN+**	**T1CN-**	**T1CN+**	**T2+N-**	**T2+N+**
	**% of patients receiving this treatment**								
**Age 35**									
No adjuvant treatment	100.0%	100.0%	13.4%	75.9%	10.1%	16.2%	8.8%	0.7%	0.8%
Only hormonal treatment	0.0%	0.0%	5.1%	0.2%	1.0%	1.2%	0.7%	0.2%	0.4%
Only chemo therapy	0.0%	0.0%	0.0%	0.0%	0.0%	0.0%	0.0%	0.0%	0.0%
Combination therapy	0.0%	0.0%	81.5%	23.9%	88.9%	82.6%	90.5%	99.1%	98.8%
**Age 60**									
No adjuvant treatment	100.0%	99.0%	6.7%	77.6%	10.6%	15.8%	10.1%	1.0%	1.1%
Only hormonal treatment	0.0%	1.0%	66.4%	6.3%	27.5%	31.0%	21.0%	7.8%	12.2%
Only chemo therapy	0.0%	0.0%	0.0%	0.0%	0.0%	0.0%	0.0%	0.0%	0.0%
Combination therapy	0.0%	0.0%	26.9%	16.2%	61.9%	53.2%	68.9%	91.3%	86.7%
**Age 75**									
No adjuvant treatment	100.0%	88.7%	0.8%	49.0%	2.9%	3.8%	3.6%	1.0%	0.7%
Only hormonal treatment	0.0%	11.3%	99.1%	50.7%	96.6%	95.8%	95.7%	96.4%	97.7%
Only chemo therapy	0.0%	0.0%	0.0%	0.0%	0.0%	0.0%	0.0%	0.0%	0.0%
Combination therapy	0.0%	0.0%	0.1%	0.3%	0.5%	0.4%	0.7%	2.6%	1.6%
**Clinically detected breast cancers**									
**Age 35**									
No adjuvant treatment	100.0%	85.2%	4.7%	42.1%	9.0%	3.0%	0.8%	0.4%	0.0%
Only hormonal treatment	0.0%	0.2%	0.2%	0.2%	0.8%	1.4%	0.2%	0.4%	0.1%
Only chemo therapy	0.0%	0.0%	0.0%	0.0%	0.0%	0.0%	0.0%	0.0%	0.0%
Combination therapy	0.0%	14.6%	95.1%	57.7%	90.2%	95.6%	99.0%	99.2%	99.9%
**Age 60**									
No adjuvant treatment	100.0%	85.6%	6.5%	50.0%	10.1%	2.9%	1.1%	0.5%	0.0%
Only hormonal treatment	0.0%	4.7%	6.7%	4.7%	22.9%	35.8%	6.4%	14.5%	4.0%
Only chemo therapy	0.0%	0.0%	0.0%	0.0%	0.0%	0.0%	0.0%	0.0%	0.0%
Combination therapy	0.0%	9.7%	86.8%	45.3%	66.9%	61.4%	92.5%	85.0%	96.0%
**Age 75**									
No adjuvant treatment	100.0%	58.6%	6.9%	44.8%	3.3%	0.6%	1.3%	0.3%	0.1%
Only hormonal treatment	0.0%	41.2%	90.3%	54.0%	96.0%	99.0%	95.5%	98.4%	94.6%
Only chemo therapy	0.0%	0.0%	0.0%	0.0%	0.0%	0.0%	0.0%	0.0%	0.0%
Combination therapy	0.0%	0.2%	2.7%	1.2%	0.6%	0.4%	3.2%	1.3%	5.3%

Appendix table 4

Appendix table 4 Discounted model estimates of the number of life years gained (LYG) and additional costs (€) per 1,000 women compared to no screening with percentage change compared to the current strategy (B50-74). The table includes the current strategy and strategies on the efficiency frontier with corresponding ICERs.

**Strategy**	**LYG**		**Additional costs (€)**		**ICER**
Biennial 50-74	54.8	-	374,763	-	Dominated
Quadrennial 60-64	12.9	-76.5%	53,050	-85.8%	4,112
Quadrennial 56-64	20.8	-62.0%	87,100	-76.8%	4,310
Quadrennial 52-64	29.5	-46.2%	126,875	-66.1%	4,572
Quadrennial 50-66	35.2	-35.8%	161,450	-56.9%	6,066
Quadrennial 50-70	38.2	-30.3%	182,304	-51.4%	6,951
Quadrennial 49-69	39.4	-28.1%	191,039	-49.0%	7,279
Quadrennial 48-68	40.6	-25.9%	199,939	-46.6%	7,416
Quadrennial 47-71	44.3	-19.2%	228,179	-39.1%	7,633
Triennial 47-71	51.4	-6.2%	294,724	-21.4%	9,372
Triennial 46-70	52.8	-3.6%	307,927	-17.8%	9,431
Triennial 44-71	57.3	4.6%	354,556	-5.4%	10,362
Triennial 44-74	58.8	7.3%	372,241	-0.7%	11,790
Triennial 43-73	60.1	9.7%	388,503	3.7%	12,510
Triennial 42-72	61.4	12.0%	405,322	8.2%	12,938
Biennial 43-73	73.1	33.4%	565,623	50.9%	13,701
Biennial 42-74	75.7	38.1%	606,977	62.0%	15,906
Biennial 41-75	78.1	42.5%	649,316	73.3%	17,641
Biennial 40-74	79.6	45.3%	676,927	80.6%	18,407
Biennial 40-76	80.4	46.7%	692,550	84.8%	19,528
Biennial 40-78	80.9	47.6%	706,655	88.6%	28,210
Annual 40-75	102.9	87.8%	1,334,950	256.2%	28,559
Annual 40-76	103.4	88.7%	1,349,662	260.1%	29,423
Annual 40-78	104.1	90.0%	1,376,779	267.4%	38,739
Annual 40-81	104.7	91.1%	1,411,796	276.7%	58,362
Annual 40-84	105.0	91.6%	1,439,916	284.2%	93,733

* The strategy with a light grey background is not on the efficiency frontier, but included because it is the current strategy. The strategy with a dark grey background is the optimal strategy based on a WTP threshold of €20,000 per QALY gained. The other strategies with a grey background are candidate strategies based on more favourable QALYs and costs compared to the current strategy.

Appendix page 7

Sensitivity analyses

Taking into account age-specific attendance rates in all modelled screening strategies decreased the number of breast cancer deaths averted, number of overdiagnoses, number of false positives, amount of QALYs gained and additional costs (Table 3). This also slightly changed which strategies were present on the efficiency frontier and the accompanying ICERs.

For the biennial 40-76 strategy, the number of breast cancer deaths averted deceased by 19.7%, the number of overdiagnoses decreased by 19.2%, the number of false positives decreased by 22..0%, the amount of QALYs gained decreased by 20.4%, and the additional costs decreased by 23.8% compared to the base case analyses. Because the decrease in additional costs is larger than the decrease in QALYs gained, the ICERs also decreased. This led to strategy biennial 40-76 no longer being the first strategy under the WTP threshold of €20,000 per QALY. This became annual screening for the ages 42 till 73.

The triennial 44-71 and triennial 44-74 strategies were not on the efficiency frontier of these sensitivity analyses. The strategies with similar additional costs and more QALYs gained or similar QALYs gained and less costs in this analyses were biennial 47-71 and triennial 45-72.

When assumptions on current adjuvant treatment use were used, the number of breast cancer deaths averted, number of overdiagnoses, number of false positives, and amount of QALYs gained were slightly lower (Table 4). On the other hand, the amount of additional costs slightly increased. In addition, the strategies which were present on the efficiency frontier and the accompanying ICERs slightly changed compared to the base case analyses.

For biennial 40-76 screening, the number of breast cancer deaths averted deceased by 1.5%, the number of overdiagnoses decreased by 1.4%, and the number of false positives decreased by 0.6% compared to the base case analyses. The amount of QALYs gained did not change, whereas the additional costs increased by 2.1%.

The triennial 44-71 and triennial 44-74 strategies were not on the efficiency frontier of these sensitivity analyses. The strategy with less additional costs and more QALYs gained was triennial 45-72.

Appendix table 5

Appendix table 5 Discounted model estimates for sensitivity analyses using alternative assumptions on current adjuvant treatment use on the number of breast cancer (BC) deaths averted, overdiagnoses, QALYs gained, and additional costs (€) per 1,000 women compared to no screening with percentage change compared to the current strategy (B50-74). The table includes the current strategy and strategies on the efficiency frontier with corresponding ICERs.

Strategy	BC deaths averted		Over-diagnoses		False positives		QALYs gained		Additional costs (€)		ICER
Biennial 50-74	5.0	-	5.8	-	89	-	62.7	-	383,034	-	Dominated
Quadrennial 60-64	1.3	-75%	1.6	-72%	18	-80%	14.5	-77%	54,129	-86%	3,732
Quadrennial 56-64	1.9	-62%	2.2	-62%	25	-72%	23.7	-62%	88,823	-77%	3,763
Quadrennial 52-64	2.5	-50%	2.7	-54%	32	-64%	33.5	-47%	129,547	-66%	4,158
Quadrennial 51-67	3.0	-40%	3.3	-43%	38	-57%	39.0	-38%	158,542	-59%	5,265
Quadrennial 50-70	3.4	-32%	3.9	-33%	44	-51%	43.4	-31%	186,294	-51%	6,366
Quadrennial 48-68	3.4	-31%	3.7	-36%	57	-36%	46.1	-26%	204,092	-47%	6,485
Quadrennial 48-72	3.8	-24%	4.4	-25%	63	-30%	48.8	-22%	223,936	-42%	7,328
Triennial 48-69	4.1	-18%	4.4	-24%	77	-13%	54.7	-13%	269,588	-30%	7,752
Triennial 48-72	4.4	-12%	4.9	-15%	83	-7%	57.0	-9%	289,087	-25%	8,518
Triennial 45-72	4.7	-5%	5.2	-10%	99	11%	63.5	1%	347,909	-9%	9,108
Triennial 43-73	5.0	1%	5.6	-4%	114	28%	67.9	8%	397,785	4%	11,364
Biennial 44-72	5.8	16%	6.2	6%	137	54%	79.3	26%	536,399	40%	12,103
Biennial 43-73	6.0	21%	6.4	11%	151	70%	82.6	32%	578,591	51%	12,781
Biennial 42-74	6.2	25%	6.7	16%	153	72%	85.4	36%	619,847	62%	14,986
Biennial 40-74	6.4	29%	6.9	19%	163	83%	89.4	43%	691,263	80%	17,655
Biennial 40-76	6.5	31%	7.2	25%	167	87%	90.2	44%	707,331	85%	19,418
Biennial 40-78	6.6	34%	7.6	30%	170	91%	90.8	45%	721,858	88%	24,382
Annual 41-75	8.0	62%	8.4	45%	253	185%	112.8	80%	1,292,534	237%	25,947
Annual 40-75	8.2	65%	8.5	47%	262	194%	115.6	84%	1,364,127	256%	26,071
Annual 40-76	8.3	66%	8.7	50%	264	197%	116.1	85%	1,379,303	260%	29,868
Annual 40-77	8.3	68%	8.9	53%	267	200%	116.5	86%	1,393,641	264%	35,235
Annual 40-79	8.4	70%	9.3	60%	271	204%	117.1	87%	1,420,068	271%	46,054
Annual 40-80	8.5	71%	9.5	63%	273	207%	117.2	87%	1,432,175	274%	67,061
Annual 40-82	8.6	72%	9.8	69%	276	210%	117.5	87%	1,454,042	280%	89,147
Annual 40-84	8.6	73%	10.1	74%	279	213%	117.6	88%	1,472,859	285%	141,295

* The strategy with a light grey background is not on the efficiency frontier, but included because it is the current strategy. The strategy with a dark grey background is the optimal strategy based on a WTP threshold of €20,000 per QALY gained. The other strategies with a grey background are candidate strategies based on more favourable QALYs and costs compared to the current strategy.

References

1. de Gelder R, Bulliard JL, de Wolf C, et al. Cost-effectiveness of opportunistic versus organised mammography screening in Switzerland. *Eur J Cancer* 2009; **45**(1): 127-38.
2. Sankatsing VDV, van Ravesteyn NT, Heijnsdijk EAM, Broeders MJM, de Koning HJ. Risk stratification in breast cancer screening: Cost-effectiveness and harm-benefit ratios for low-risk and high-risk women. *Int J Cancer* 2020; **147**(11): 3059-67.
3. Netherlands Comprehensive Cancer Organisation. https://iknl.nl/ (accessed 07-05 2021).
4. de Koning HJ, Boer R, Warmerdam PG, Beemsterboer PM, van der Maas PJ. Quantitative interpretation of age-specific mortality reductions from the Swedish breast cancer-screening trials. *J Natl Cancer Inst* 1995; **87**(16): 1217-23.
5. Tabár L, Vitak B, Chen HH, et al. The Swedish Two-County Trial twenty years later. Updated mortality results and new insights from long-term follow-up. *Radiol Clin North Am* 2000; **38**(4): 625-51.
6. Nyström L, Andersson I, Bjurstam N, Frisell J, Nordenskjöld B, Rutqvist LE. Long-term effects of mammography screening: updated overview of the Swedish randomised trials. *Lancet* 2002; **359**(9310): 909-19.
7. Bjurstam N, Björneld L, Warwick J, et al. The Gothenburg Breast Screening Trial. *Cancer* 2003; **97**(10): 2387-96.
8. Early Breast Cancer Trialists' Collaborative Group. Effects of chemotherapy and hormonal therapy for early breast cancer on recurrence and 15-year survival: an overview of the randomised trials. *Lancet* 2005; **365**(9472): 1687-717.
9. StatLine. Levensverwachting; geslacht,leeftijd (per jaar en periode van vijf jaren). 09-06-2020 2020. https://opendata.cbs.nl/statline/#/CBS/nl/dataset/37360ned/table?fromstatweb (accessed 14-04 2021).
10. Geuzinge HA, Bakker MF, Heijnsdijk EAM, et al. Cost-Effectiveness of Magnetic Resonance Imaging Screening for Women With Extremely Dense Breast Tissue. *JNCI: Journal of the National Cancer Institute* 2021.
11. Netherlands Comprehensive Cancer Organisation. Monitor bevolkingsonderzoek borstkanker 2017/2018: IKNL, 2019.
12. Polder JJ, Barendregt JJ, van Oers H. Health care costs in the last year of life--the Dutch experience. *Soc Sci Med* 2006; **63**(7): 1720-31.
13. Bakx P, O'Donnell O, van Doorslaer E. Spending on Health Care in the Netherlands: Not Going So Dutch. *Fiscal Studies* 2016; **37**(3-4): 593-625.
14. Versteegh MM, Vermeulen KM, Evers SMAA, de Wit GA, Prenger R, Stolk AE. Dutch Tariff for the Five-Level Version of EQ-5D. *Value Health* 2016; **19**(4): 343-52.
15. van Luijt PA, Fracheboud J, Heijnsdijk EA, den Heeten GJ, de Koning HJ, National Evaluation Team for Breast Cancer Screening in Netherlands Study G. Nation-wide data on screening performance during the transition to digital mammography: observations in 6 million screens. *Eur J Cancer* 2013; **49**(16): 3517-25.
16. Skaane P, Hofvind S, Skjennald A. Randomized Trial of Screen-Film versus Full-Field Digital Mammography with Soft-Copy Reading in Population-based Screening Program: Follow-up and Final Results of Oslo II Study. *Radiology* 2007; **244**(3): 708-17.
17. Perry NM, Patani N, Milner SE, et al. The impact of digital mammography on screening a young cohort of women for breast cancer in an urban specialist breast unit. *Eur Radiol* 2011; **21**(4): 676-82.
18. Domingo L, Hofvind S, Hubbard RA, et al. Cross-national comparison of screening mammography accuracy measures in U.S., Norway, and Spain. *Eur Radiol* 2016; **26**(8): 2520-8.

19. Sankatsing VD, Heijnsdijk EA, van Luijt PA, van Ravesteyn NT, Fracheboud J, de Koning HJ. Cost-effectiveness of digital mammography screening before the age of 50 in The Netherlands. *Int J Cancer* 2015; **137**(8): 1990-9.
20. CZ. Tarieventool. https://www.cz.nl/service-en-contact/zoek-tarieven (accessed 07-05 2021).
21. de Haes JC, de Koning HJ, van Oortmarssen GJ, van Agt HM, de Bruyn AE, van Der Maas PJ. The impact of a breast cancer screening programme on quality-adjusted life-years. *Int J Cancer* 1991; **49**(4): 538-44.
22. Stout NK, Rosenberg MA, Trentham-Dietz A, Smith MA, Robinson SM, Fryback DG. Retrospective Cost-effectiveness Analysis of Screening Mammography. *JNCI: Journal of the National Cancer Institute* 2006; **98**(11): 774-82.
